# Using an experience-based co-design approach to develop strategies for implementing an intravenous iron intervention to treat moderate and severe anemia in pregnancy in Malawi

**DOI:** 10.1186/s43058-024-00661-1

**Published:** 2024-11-15

**Authors:** Elisabeth Mamani-Mategula, Naomi Von-Dinklage, Hana Sabanovic, Ebony Verbunt, Khic-Houy Prang, Effie Chipeta, Lucinda Manda-Taylor

**Affiliations:** 1 Department of Health Systems and Policy, The Kamuzu University of Health Sciences, Blantyre, Malawi; 2 School of Population and Global Health, Centre for Health Policy, The University of Melbourne, Melbourne, Australia; 3 Population Health and Immunity Division, The Walter and Eliza Hall Institute of Research, Melbourne, Australia

**Keywords:** Community engagement, Experience-based co-design, Implementation strategies, Anemia, Intravenous iron, Pregnant women, Antenatal care

## Abstract

**Background:**

In low- and middle-income countries, women experiencing anemia during pregnancy are recommended to take 30 mg to 60 mg of oral iron daily throughout pregnancy. However, oral iron tablets are often poorly tolerated and slow in correcting anemia, resulting in low adherence, prolonged anemia, and increased risk of adverse maternal and fetal outcomes. An alternative to oral iron is intravenous (IV) iron, commonly used in high-income countries to restore the body’s iron stores rapidly. A randomized controlled trial was conducted to investigate the effectiveness and safety of IV iron compared to standard-of-care oral iron supplementation for pregnant women with moderate and severe anemia in the third trimester in Malawi (REVAMP-TT). Using an experience-based co-design approach, our study aimed to identify barriers and facilitators to IV iron use to treat anemia in pregnancy in the primary healthcare system of Malawi, and develop mitigating strategies for the successful implementation of REVAMP-TT.

**Methodology:**

The co-design process involved two phases: i) We conducted an information-gathering exercise to identify barriers and facilitators to IV iron use to treat anemia in pregnancy in the primary healthcare system of Malawi. We interviewed key informants (n = 53) including the policymakers, government partners, healthcare managers, and healthcare providers. We also gathered previous research findings from a formative qualitative study on the perceptions and experiences of IV iron treatment for pregnant women experiencing anemia in Malawi (n = 29). ii) We conducted two co-design workshops with end-users (n = 20) and healthcare providers (n = 20) to confirm and identify the key barriers and facilitators and developed mitigating strategies to inform the successful implementation of the REVAMP-TT trial. We mapped the emerging barriers to the Consolidated Framework for Implementation Research 2.0 (CFIR 2.0) and matched the mitigating strategies to the corresponding Expert Recommendations for Implementing Change (ERIC) compilation.

**Results:**

The following were identified as key barriers to IV iron use to treat anemia in pregnancy in the primary healthcare system of Malawi: the cost of IV iron, the lack of available resources and knowledge, local attitudes including myths and misconceptions about IV iron and keeping pregnancy a secret, local conditions, the lack of political will and buy-in from high-level leaders, the lack of capability of healthcare providers to deliver IV iron, and the lack of male involvement to support pregnant women's access to antenatal care. The proposed strategies to mitigate the barriers for the successful implementation of the REVAMP TT trial included providing financial strategy, developing stakeholder relationships, training and educating stakeholders, supporting clinicians, and engaging end-users.

**Conclusion:**

The use of the experience-based co-design approach in our study provided a valuable method to expose the potential barriers and facilitators to IV iron use and develop mitigating strategies to successfully implement the REVAMP-TT trial. Engaging both the key informants and end users promoted ownership and consensus among stakeholders and ensured a collaborative environment for sharing deeply rooted real-world experiences and insights. Not only do these findings address the needs of this study, but they also, lay a groundwork for the possible integration of IV iron into routine care in Malawi and provide knowledge for policymakers to make informed decisions on the management of anemia in the primary healthcare systems of Malawi.

Contributions to the literature

- Our study serves as a methodologic reference for other researchers seeking to utilize co-design methodologies as an effective, feasible approach to identify implementation barriers and corresponding strategies to promote the use of clinical interventions.
- Our study identified key barriers to IV iron use for treating anemia in pregnancy, including cost, resource limitations, knowledge gaps, local attitudes, and lack of stakeholder engagement. Strategies to address these include financial strategy, stakeholder relationship building, training, clinician support, and end-user engagement. These insights can guide healthcare providers, the Ministry of Health, government partners, and other stakeholders in improving maternal health outcomes in Malawi.
- In our study, we realized that barriers and facilitators exist across different health system levels, organized according to the CFIR framework. This underscores the need for tailored strategies according to the levels to address challenges for effective implementation of health intervention.

## Background

Anemia during pregnancy is a global health problem. The World Health Organization (WHO) reports that nearly half of pregnant women experience anemia globally; 18% are from high-income countries (HICs), and 35–75% are from low- and middle-income countries (LMICs) [1, 2]. The prevalence rate of anemia during pregnancy in Malawi is 41.8% (Hb < 11.0 g/dl) [3]. The WHO recommends the daily uptake of oral iron tablets for managing anemia in pregnancy [4–7]. However, oral iron tablets are often poorly tolerated and adhered to because of significant gastrointestinal effects, tedious daily intake, frequent need to visit antenatal care clinics for refills, and issues of low supply [4, 8–10].

A potential alternative to treat moderate and severe anemia during pregnancy when oral iron is poorly tolerated is intravenous (IV) iron. Recent studies have demonstrated that IV iron is superior to oral iron in managing moderate and severe anemia during pregnancy, as it quickly replenishes the body's iron stores after one or two infusions [7, 11–13]. Although IV iron is routinely used in some HICs for the treatment of anemia during pregnancy, there is limited research on the applicability and transferability of this intervention to LMICs.

Past research has shown that clinical interventions are more readily adopted into routine practice if end-users and healthcare providers are involved in all stages of planning and implementation of interventions [14]. Implementation science research is crucial and plays a significant role in bridging the gap between research and practice. It ensures that all relevant stakeholders are engaged and that interventions are effectively and efficiently translated into real-world settings to improve the quality and effectiveness of health services [15]. The systematic application of implementation science methodologies and frameworks can aid in understanding different barriers and facilitators, key moments and experiences along the end-user journey, behaviors, and needs of both healthcare providers and end-users across different contexts for clinical interventions [16].

### REVAMP and REVAMP-TT

In Malawi, a Randomized controlled trial of the Effect of intravenous iron on Anemia in Malawian Pregnant women (REVAMP) was conducted to compare the effect of IV iron with the standard-of-care, oral iron for the treatment of moderate and severe anemia in the second trimester [17]. This study was managed by the Training and Research Unit of Excellence (TRUE) and implemented by the study staff at the research site infrastructure. Results showed that IV iron, compared to oral iron, did not reduce anemia prevalence at 36 weeks gestation. However, results showed that pregnant women who received IV iron in the second trimester had a lower prevalence of anemia than those who received oral iron four weeks post-IV iron infusion, which suggested that the IV iron was given too early to see its advantages sustained on the mother at 36 weeks and by delivery [18]. A Randomized controlled trial of the Effect of intraVenous iron on Anemia in Malawian Pregnant women in the Third Trimester (REVAMP-TT) was conducted to determine whether a single dose of ferric carboxymaltose (FCM) compared to oral iron would be effective in treating moderate or severe anemia and improve maternal and child health outcomes at delivery [19]. The REVAMP-TT trial was implemented in primary healthcare facilities involving government healthcare providers to represent real-world settings and understand the applicability and transferability of the intervention to the local context.

### REVAMP-IS

To support the implementation of an IV iron intervention in the REVAMP-TT trial, we performed an Implementation Science research program called REVAMP-IS. An associated protocol paper has been previously published [20]. Briefly, the implementation research program was structured into three phases: i) Formative research involving context assessment of the Malawian health system, ii) Developing implementation strategies to support the uptake and delivery of IV iron intervention, iii) Evaluation of the implementation of IV iron intervention, and strategies [20]. This paper describes the REVAMP-IS research program's findings from phases 1 and 2.

#### Aims of study

In this paper, we outline the application of an experience-based co-design (EBCD) approach to i) identify key touchpoints (critical moments where individuals interact with healthcare services that shape their experiences) influencing the barriers and facilitators to IV iron use to treat anemia in pregnancy in the primary healthcare system of Malawi, and ii) develop mitigating strategies for the successful implementation of REVAMP-TT trial. The findings can also inform the integration of IV iron into Malawi’s primary healthcare system if the trial demonstrates its effectiveness, thereby improving the translation of research into practice.

### Methodology

#### Study setting

We conducted the REVAMP-TT trial and the implementation research program in the eight primary health facilities of the Zomba district in Malawi, covering both rural (Likangala, Bimbi, Lambulira, and Domasi) and urban (City Clinic, Sadzi, Matawale, and Naisi) areas. These health facilities served as recruitment locations for eligible pregnant women participating in the REVAMP-TT trial and accessing antenatal care services, including screening and treating anemia.

#### Experience-based co-design

**Fig. 1:**
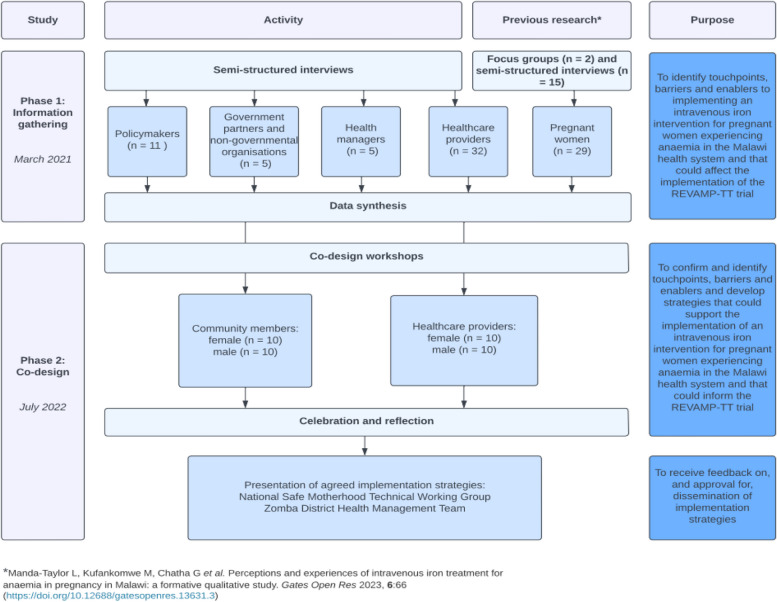
Experience-based co-design orientation and collaborative change processes

We used an experience-based co-design (EBCD) approach to gather insights from different stakeholders on the implementation of the REVAMP-TT trial and routine antenatal care. EBCD refers to a collaborative and participatory creative process of co-designing healthcare services with end-users, healthcare providers, and policymakers through an equal partnership approach, which ensures the development of more engaging and satisfying interventions for potential end-users [21, 22]. This approach minimizes the waste of funds and resources by designing and developing interventions and implementation strategies that consider local needs and address potential barriers from the outset. The process involved two phases: i) An information-gathering phase to identify key touchpoints influencing the barriers and facilitators to IV iron use to treat anemia in pregnancy in the primary healthcare system of Malawi, and ii) Co-design workshops were held with end-users and healthcare providers to share the barriers and facilitators, identify priorities, and develop mitigating strategies for successfully implementing the REVAMP-TT trial. We based our EBCD workshops on inclusivity, participation, respectfulness, adaptation, and outcomes to ensure authentic engagement and power sharing amongst participants [21, 22]. Figure 1 illustrates our experience-based co-design orientation, the collaborative change processes, and the associated number of participants in each step.

- Phase 1: Information gathering

The aim of Phase 1 was to gather information by identifying the touchpoints influencing the barriers and facilitators to IV iron use to treat anemia in pregnancy in the primary healthcare system of Malawi. This was conducted in two steps*.*

- Step 1a) Gathering touchpoints influencing the barriers and facilitators to IV iron use to treat anemia in pregnancy in the primary healthcare system of Malawi

The Consolidated Framework for Implementation Research 2.0 (CFIR 2.0) defined in Appendix 1 guided the development of the interview guide, focusing on the domains of innovation, individuals, outer setting, inner setting, and process. We opted to use CFIR 2.0 because it acknowledges that the effectiveness of interventions implemented within a health system is influenced by the interaction of environmental/policy within the larger healthcare system and by organizational and individual-level factors. We wanted to ensure that a comprehensive range of the potential barriers and facilitators to IV iron use to treat anemia in pregnancy in the primary healthcare system of Malawi were explored. Our focus was on gathering insights on both patient and provider factors within the Malawian health system. We piloted the tool guide with 2 healthcare providers and one policymaker from Blantyre District Health Office, none of whom were part of the study. We then conducted debriefings to reflect on the data collection process and refine the tool guide.

We (EMM) conducted key informant interviews (KIIs) including policymakers, government partners, health managers, and healthcare providers to understand perspectives on the use of IV iron to treat anemia in pregnancy and their experiences working within the Malawian health system. We purposively selected key informants directly involved in antenatal care services. Snowball sampling was also used to identify further potential participants. We invited them through emails enclosed with the participant information sheet and followed them up with phone calls. In total, 58 participants were invited, 53 accepted (91% participation rate) and 6 declined as they were engaged in other activities. We conducted one-on-one interviews in English which lasted an average of 45 min.

- Step 1b) Data analysis

Interviews were audio-recorded and then transcribed by a professional transcription service. We (EM, LMT, NVD) independently developed the codebook and coded the same three transcripts to identify the emerging themes on the barriers and facilitators to IV iron use to treat anemia in pregnancy in the primary healthcare system of Malawi. These were compared and discussed, leading to an agreed-upon codebook. EM coded the rest of the transcripts using NVivo Version 12. We applied an inductive approach to flexibly and naturally capture the emerging themes without being constrained to the CFIR framework. The emerging themes were then organized and systematically mapped to the relevant CFIR constructs for a clear interpretation of the barriers and facilitators to IV iron use to treat anemia in pregnancy. This approach balanced the use of a predefined framework with the emergence of data-driven themes, ensuring robustness, rigor, and openness in the findings.

- Step 2. Gathering healthcare providers’ and end-user experiences

To complement the above findings, we identified additional key themes on the touchpoints influencing the barriers and facilitators to the use of IV iron from a previously published study that explored the perceptions and experiences of IV iron among pregnant women and healthcare providers participating in the REVAMP trial [23].

We synthesized the findings from the KIIs and the previous qualitative study by reviewing the key themes to identify overlaps and differences. We then mapped the themes to CFIR 2.0. The synthesized themes were used as the basis for discussion to understand a pregnant woman’s antenatal care journey in the co-design workshops. The participants from the KIIs were not the same participants who participated in the co-design workshops.

- Phase 2: Co-design workshops

We (EMM, EC, and LMT) conducted two separate co-design workshops. The first workshop included community members, pregnant women with experience receiving IV iron, caregivers, mothers-in-law, spouses, village health committee members, and village heads. The second workshop included healthcare providers: doctors, clinicians, nurses and midwives, health surveillance assistants, laboratory technicians, pharmacists, and data clerks. The co-design workshops aimed to discuss the touchpoints influencing the barriers and facilitators to IV iron use to treat anemia in pregnancy in the primary healthcare system of Malawi as identified in Phase 1. Together, we selected the prioritized barriers and developed mitigating strategies for the successful implementation of REVAMP-TT.”

We conducted separate co-design workshops with the community members and healthcare providers to address concerns about power dynamics. We aimed to create an environment where each group could express themselves freely and without influence, ensuring their voices were heard. This approach allowed for more in-depth conversations, enabling each group to share their unique experiences and perspectives. The insights gathered from both groups were thoughtfully combined and synthesized to accurately represent the needs of all stakeholders involved.

- Step 3. Recruitment of community members and healthcare providers

Community members: Participants were systematically recruited using the REVAMP trial register. This is a database of all the women who experienced anemia during pregnancy and received IV iron in their second trimester. We visited them in their homes to personally invite them to participate in the co-design workshop and asked them to come along with either their spouses, guardians, or local leaders. All the approached participants agreed to participate.

Healthcare providers: The research coordinator provided a list of healthcare providers from the facilities involved in the REVAMP-TT trial. Twenty participants were purposively recruited through the District Nursing Officer for Zomba, and we invited them via telephone and email. All prospective participants agreed to participate in the study after being transparently informed about the community.

Participants likely chose to participate in the co-design workshops due to minimal associated risks, as no samples would be collected or interventions administered. The National Health Sciences Research Committee in Malawi recommends that all human subjects research offer participants US$10 per study visit as compensation for costs which may have also influenced their decision.

- Step 4: Jointly identify priority touchpoints influencing the barriers and facilitators to IV iron use to treat anemia in pregnancy in the primary healthcare system of Malawi

We (EMM, EC, and LMT) commenced the co-design workshops with an overview of the REVAMP-TT trial, the implementation research program, and the purpose of the workshops. We then described the touchpoints influencing the barriers and facilitators to IV iron use to treat anemia in pregnancy in the primary healthcare system of Malawi as identified in Phase 1. Through group discussions and consensus, we together narrowed down the priority barriers that were likely to impact the use of the IV to treat anemia in pregnancy. During the group discussions, the community members were divided into two gender-based groups (men/women) to address power dynamics and respect for cultural-gender considerations, contrasting with the workshop for healthcare providers, where both males and females were combined. We conducted the workshops in Chichewa, the local language of Malawi.

- Step 5: Jointly develop mitigating strategies for the successful implementation of REVAMP-TT

After identifying the key barriers, participants regrouped to discuss the main facilitators that would support the use of IV iron in the REVAMP-TT trial. This discussion helped in developing the strategies aimed at overcoming the barriers. They then selected key strategies that would mitigate the barriers and ensure the successful implementation of the REVAMP-TT trial in the primary healthcare system of Malawi. A representative from each group shared their conclusions with the broader group.

### Data analysis of steps 4 and 5

We (EMM, LMT) recorded data from the co-design workshop through short notes, flip charts, sticky notes, and audio. We (EMM, LMT, EC), together with the participants, debated and deliberated on the prioritized barriers and implementation strategies proposed by the participants. We discussed and selected the most important barriers and strategies based on consensus decision-making, feasibility, and the time required to implement the strategies. Although we did not use the Expert Recommendations for Implementing Change (ERIC) compilation to guide our discussions in the co-design workshops, it was used in the data analysis because the strategies that emerged from participants aligned with the structured context of the ERIC tool. ERIC is a compilation of implementation strategies, and it includes 73 defined implementation strategies clustered into nine content areas [24]. Consequently, we (EMM and HS) together mapped the emergent implementation strategies to the ERIC compilation.

- Step 6: Reflections and celebrations

We held meetings with the REVAMP-TT TRUE research team, policymakers, and district health managers for a joint discussion on the findings from the co-design workshops, and the selection of implementation strategies. We presented the outcomes from the co-design workshops at the Malawi National Safe Motherhood Technical Working Group (SMTWG) (a group that serves various functions, including policy development, program planning, and implementation for maternal health at the national level), the Zomba District Health Management Team (DHMT) and the community for feedback, insights, recommendations, and dissemination purposes.

### Consent and ethics clearance

We received ethics approval (P.08/20/3114) from the University of Malawi’s College of Medicine Research and Ethics Committee. Permission to collect data from the health facilities was obtained from the Zomba District Health Office (DHO) research committee. Written informed consent was provided in Chichewa (the local language) or English according to the participants’ language preference. Literate participants provided a signature on the consent form, and illiterate participants provided a thumbprint. We informed all participants that confidentiality would be maintained and no personal details would be divulged. We also explained to the participants that their involvement in the research was voluntary and that withdrawal was permitted at any time without personal consequences. Finally, we provided all participants with a copy of their written consent form and reimbursed them for their time and travel expenses per Malawi’s national participant remuneration guidelines directed by the Malawi government through the National Health Sciences Research Committee (NHSRC). All human subjects research should provide study participants with US$10 per study visit as compensation for costs [25]. The compensation covers transportation, lunch, and lost wages or missed economic opportunities resulting from participating in the study [26]. A minimum compensation of $10 per study visit was established to prevent exploitation [26] and to show appreciation and gratitude for participation in the study [27].

## Results

### Participant demographics

Table 1 shows the demographic characteristics of participants for the key informant interviews (KIIs) and co-design workshops 1 and 2.

**Table 1:** Key informant interviews and co-design workshops participants' demographic characteristics

		P**hase 1**: **Information gathering**	**Phase 2:** **Co-design workshops**	
**Variable**		**Key informant interviews**	**Community members**	**Healthcare providers**
**Gender**	*Male*	*23*	10	10
	*Female*	*30*	10	10
**Age**	*20–29*	*6*	6	2
	*30–39*	*18*	5	8
	*40–49*	*24*	5	6
	*50–60*	*5*	4	4
**Marital status**	*Married*	*41*	12	13
	*Unmarried*	*7*	1	6
	*Divorced/Widowed*	*5*	7	1
**Education**	*Illiterate*	*-*	2	-
	*Primary*	*-*	11	*-*
	*Secondary*	*-*	7	3
	*Tertiary*	*53*	-	17
**Occupation**	*Policymaker*	*11*	-	-
	*Health manager*	*5*	*-*	*-*
	*Government partner*	*5*	-	-
	*Healthcare providers*	*32*		
	*Doctor*	*-*	-	2
	*Nurse*	*-*	-	5
	*Clinical officer*	*-*	-	3
	*Community Midwife*			3
	*Patient attendant*	*-*	-	3
	*Lab technician*	*-*	*-*	2
	*Data clerk*	*-*	*-*	2
**Community role**	*Wife (participants in REVAMP trial)*		4	
	*Mother-in-law*	*-*	2	-
	*Elderly*		2	
	*Village head*	*-*	3	-
	*Village health committee*		3	
	*Husband*	*-*	4	-
	*Father/father-in-law*	*-*	2	-

## Barriers mapped to CFIR 2.0 and corresponding strategies mapped to the ERIC compilation

We identified key barriers that could affect the use of IV iron to treat anemia in pregnancy in primary healthcare of Malawi, corresponding to the following CFIR 2.0 domains: innovation, inner setting, outer setting, individual, and implementation process. These include the cost of IV iron, lack of available resources and knowledge, local attitudes including myths and misconceptions of IV iron, local conditions affecting access to antenatal care, lack of political will and buy-in from high-level leaders, lack of capability from healthcare providers to deliver IV iron and lack of male involvement to support pregnant women accessing care. The proposed strategies aligned with the following ERIC domains: providing financial strategies, developing stakeholder relationships, training and educating stakeholders, supporting clinicians, and engaging end users and the community. Table 2 shows how we mapped the key barriers to the CFIR framework and matched them to the selected strategies in the ERIC compilation.

**Table 2:** Outlining the priority barriers identified in phase 1 mapped to CFIR 2.0 and corresponding strategies identified through a co-design process in phase 2 mapped to the ERIC compilation

CFIR 2.0 Domains	Phase 1: Information gathering		Phase 2: Co-design workshops		
	**Barriers from key informant interviews priotised in the co-design workshops**	**Barriers from qualitative study prioritized in the co-design workshops** [23]	**Strategies identified from the facilitators**	**Action points**	**ERIC tool Content area-strategy**
Innovation domain	Cost of IV iron	Cost of IV iron	IV iron should be included in healthcare budget	Prioritized. REVAMP-TT trial to cover treatment costs	Financial strategies
Inner setting domain	Lack of knowledge and information about anemia amongst healthcare providers		Development and dissemination of IV iron information	Prioritized: To conduct education meetings and outreach activities to healthcare providers	Train and educate stakeholders
	Lack of available resources: human resources	Relative disadvantage: time to deliver IV iron	Recruitment of additional healthcare providers	Prioritized: REVAMP-TT to recruit additional healthcare providers for the trial	Financial strategies
	Lack of available resources: private spaces, equipment	Lack of available resources	Provision of additional resources	Prioritized: Equipment and resources for treatment to be provided by REVAMP-TT	Financial strategy
	Workplace culture is not patient-centered		Improve healthcare providers’ attitudes	Prioritized: To provide incentives or counselling to healthcare providers	Support clinicians
Outer setting domain	Local attitudes: myths and misconceptions relating IV iron to vampirism and satanism	Procedures related to satanism and vampirism	Community engagement	Prioritized: Mass media to reach large numbers of people in the community to dispel myths and misconceptions and increase demand for IV iron	Engage consumers
	Local conditions e.g. lack of transport and difficulty accessing health services		Strengthening the referral systems	Not prioritized as beyond scope of study	Financial strategies
	External pressure: culturally keeping pregnancy as a secret	Cultural beliefs that IV iron will cause miscarriage	Community sensitization and awareness	Prioritized: Mass media to reach large numbers of people in the community to increase demand for IV iron	Engage consumers
Individual domain	High level leaders: lack of political will and buy-in from the policy makers		Stakeholder engagement through SMTWG and district health management team	Prioritized: Attend advisory SMTWG and district health management team meetings to provide input and elicit recommendations for the implementation of REVAMP-TT trial	Develop stakeholder relationships
	Innovation deliverers: need for training to deliver IV iron		Training and orientation of healthcare providers for anemia screening and IV iron preparation and administration	Prioritized: To revise REVAMP-TT trial training protocol including roles of healthcare providers	Support clinicians
	Opinion leaders: Lack of male involvement in antenatal care hinders pregnant women receiving IV iron	Lack of male participation in antenatal care	Identify male champions	Prioritized: Men to promote antenatal care and pregnant women to consult their husbands before enrolling in REVAMP-TT trial	Engage consumers
	Need: Lack of knowledge on anemia screening and management amongst pregnant women		Community education and sensitisation	Prioritized: To develop information education and communication materials to provide knowledge to pregnant women on anemia screening and management	Train and educate stakeholders
Implementation process domain	Engage: Lack of stakeholder’s engagement		Stakeholder engagement	Prioritized: To conduct stakeholder engagement meetings with SMTWG and district health management team,	Develop stakeholder relationships
	Reflecting and evaluating emphasis		Dissemination of research findings	Prioritized: To present and review our findings with the SMTWG, district health management team and community members	Develop stakeholder relationship

*CFIR2.0* Consolidated Framework for Implementation Research 2.0, *ERIC* Expert Recommendations for Implementing Change, *SMTWG* Safe Motherhood Technical Working Group, *DHMT* District Health Management Team

**Fig. 2:**
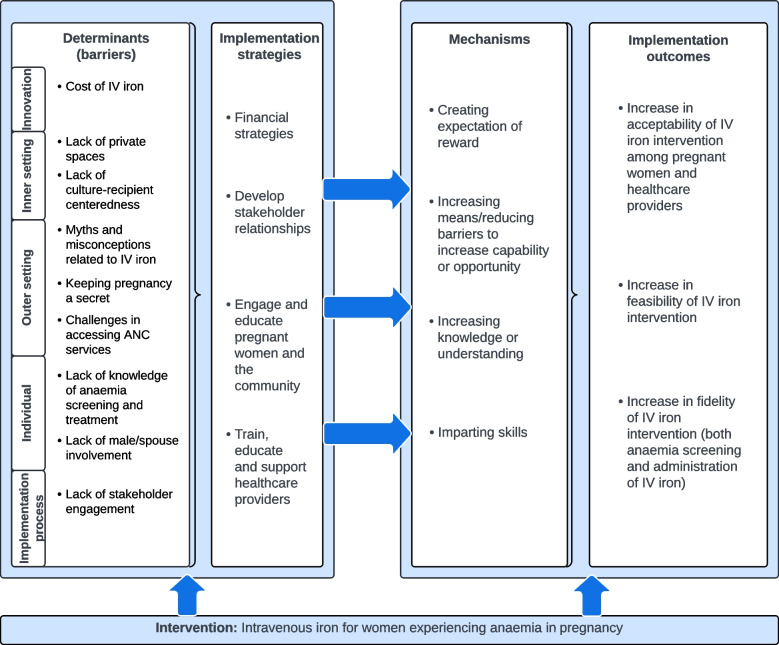
Key barriers mapped to selected implementation strategies in an implementation research logic model

To illustrate our findings, we mapped the key barriers and selected implementation strategies in an implementation research logic model shown in Fig. 2. We envisaged the implementation strategies will increase the feasibility and acceptability of an IV iron intervention as part of the REVAMP-TT trial via several mechanisms.

A. Innovation domain
  1. The cost of using IV iron in Malawi

The use of IV iron is a novel intervention in Malawi. KII participants reported concerns about the associated costs related to IV iron administration. They indicated that Malawi has a limited supply of essential drugs, such as oral iron tablets and other resources, and IV iron would be costly.

“A woman can start antenatal care visits and deliver without receiving a dose of ferrous sulfate. Are we going to manage with the expensive IV iron? Most often, iron tablets are out of stock. Sometimes, clients are even advised to buy their drugs. There are no hemocues; we lack BP [blood pressure] machines, weighing scales, and even IV drug-giving sets. We cannot afford this drug.” (Co-design workshop 2: nurse-03).

B. Outer setting domain
  1. Local conditions

Participants from the KII questioned the intervention's transferability from HICs to Malawi because of the challenges in accessing antenatal care services. Pregnant women have limited means of transport and must travel long distances over poor terrain. This discourages pregnant women from seeking routine check-ups, including for screening and treatment of anemia, even though it is important to perform routine check-ups to ensure timely diagnosis and treatment of anemia.

“Women feel it is so tiresome to come for antenatal care every month because of the long distances they have to endure to travel to a health facility, hence a missed opportunity for diagnosing anemia. They normally come when they are nearing delivery. Roads are dangerous during the rainy season, and when vegetation grows thick, they cannot travel to the facility alone. If they find no guardian, they stay home.” (KII: policy maker-02).

2. Local attitudes

### Myths and misconceptions about IV *iron*

Participants from both the KII and the co-design workshops mentioned that myths and misconceptions about IV iron would likely discourage pregnant women from participating in the REVAMP-TT trial and receiving IV iron. Identified myths and misconceptions included relating IV iron to the COVID-19 vaccine, satanism, and vampirism, mistrusted motives on routine follow-ups and invasive medical procedures, as well as fear of pain.

“We [pregnant women] are poor in the community. Nobody expects a car to come to our house. When they see the organization's vehicle coming to our house for follow-up visits, they call on each other to come and witness that we have joined Satanism. Some say that we have sold our babies to satanism because of famine to receive the allowance we get from the project and buy food.” (Co-design workshop 1: pregnant woman-04).

“IV iron looks like blood, and that would be associated with vampirism. Additionally, anything about injection currently, is related to the COVID-19 vaccine, which people are against. They would also think you are injecting something unknown into their bodies to make them unable to conceive again.” (Co-design workshop 1: pregnant woman-02).

### Keeping pregnancy a secret

Participants from the co-design workshops mentioned that cultural factors and personal preferences to hide pregnancy influence pregnant women's ability to seek antenatal care services. In many remote cultures in Malawi, women are encouraged to conceal their pregnancy until the second trimester due to misconceptions that public knowledge will lead to miscarriage. This practice contributes to delayed and infrequent monitoring of the health of both the mother and the child, including the screening and management of anemia.

“Most of them [pregnant women] think they will lose the baby if they announce their pregnancy. Pregnant women used traditional medicine to keep the pregnancy without visiting the hospital. They will only come when they know they are due soon. How can anemia be treated at this stage when it is supposed to be diagnosed early?” (KII- policy maker-04).

III. Inner setting domain
  1. Lack of available resources

### Lack of private space

Inadequate space in the health facilities was reported as a barrier to providing the IV iron intervention. Given the need for privacy for anemia screening and IV iron administration, healthcare providers complained that there would not be enough room to conduct these procedures.

“There is also inadequate space at the health facilities. For example, there are not enough treatment rooms or guardian shelters. Unless you bring the [privacy] screens, maybe we can divide the postnatal ward into a separate room.” (Co-design workshop 2: clinician-07).

### Shortage of human resources

Understaffed health facilities were also reported as a barrier to timely access to antenatal care because IV iron administration requires at least 15–30 min and an additional 30 min for monitoring. Inadequate numbers of healthcare providers were linked to increased workload and contributed to long queues and significant waiting times for pregnant women.

“The only thing I see in this [IV iron program] is that nurses and midwives are already overwhelmed with work, like going to a health facility for antenatal care, where approximately 300 or 400 women are being attended to by one or two nurses. This then needs to come with reprogramming because I see that for it to be translated on the ground is an issue of human resources.” (KII: policy maker-03).

### Culture-recipient centeredness

Women who participated in the co-design workshop strongly reported that healthcare providers’ poor attitude and inability to provide clear explanations of procedures, such as blood sample collection and drug injection, create fear and anxiety in the women. Women do not feel free to share their concerns and experiences with healthcare providers. This erodes their trust and willingness to attend antenatal care services, hence missing appointments for routine anemia screening and timely treatment.

“Nurses shout a lot, and with this, we [pregnant women] don’t look forward to meeting them in the next appointment, so we just stay home and miss the appointment. I still remember what a nurse said accusing me of going for labor and delivery approximately 1:00 a.m., yet no one chooses when the best time is to go until labor starts” (Co-design workshop 1: pregnant woman-04).

D. Individual characteristics domain
  1. Innovation recipient

### Lack of knowledge of *anemia* screening and treatment

KII showed that the introduction of a new medical intervention involving drawing blood samples would make it difficult for pregnant women to willingly participate in the REVAMP-TT trial and accept the IV iron intervention. Pregnant women lacked information about the benefits, effects, and importance of IV iron, and participants in the co-design workshops confirmed this.

“Pregnant women complain that nine months is a long time to visit the health facility every month. As such, they stay at home and ignore headaches and fatigue. They feel it will go away, not knowing that it could be anemia and will need treatment. By the time they reach the health facility at seven months of pregnancy, the problem is unmanageable. Maybe they don’t know that anemia is dangerous”. (KII: district health manager -05).

### Lack of male/spouse involvement

While healthcare providers encourage men to accompany their wives for antenatal care visits, pregnant women in the co-design workshops highlighted that they lacked this support from their spouses. A lack of male involvement was mentioned as a hindrance to women's ability to decide whether or not to participate in the REVAMP-TT trial. Men in the co-design workshops acknowledged the fear of being tested for HIV/AIDS and work commitments as reasons why they do not attend their wife’s antenatal care appointments.

“If men accompany us [pregnant women] to antenatal care, we feel quickly more comfortable to decide on interventions like these [IV iron] knowing that they agree with the advice we get from the hospital.” (Co-design workshop 1: female participant-03).

2. Innovation delivery

### Training for anemia screening and administration of IV iron

Healthcare providers confirmed that they do not currently administer IV iron in health facilities and will therefore, need training on drug preparation, administration, and storage.

“It’s a new drug; no one [healthcare providers] is familiar with it, and we don’t know how to prepare and administer IV iron. Does it have any benefits or side effects? Can it be kept in cabinets, at room temperature, or in refrigerators if a lower temperature is needed? All these are the questions that we have.” (Co-design workshop 2: Pharmacist-02).

E Implementation process domain

1. Engagement

A lack of stakeholder engagement was described as the main barrier to buy-in and acceptability of the IV iron intervention. The KIIs stressed the importance of engaging all stakeholders, including pregnant women, the community, healthcare providers, health managers, policymakers, and government partners, at every phase of the REVAMP-TT trial.

2. Reflecting and evaluating

Participants in the KII and co-design workshops stressed the need to implement evidence-based interventions. They emphasized that this can be achieved only if researchers share their findings and outcomes across all relevant stakeholders’ platforms, involving the policymakers, government partners, healthcare providers, and the community.

“For communities, change is something that may meet resistance, or it may be taken on board depending on how the community would perceive it, so once you are bringing a change, it’s good to understand the leadership structures of the community and involve the community. You can do it through the chiefs because they have different structures and they could say, maybe you will meet with such a group. Right now, I know there are community action groups who can agree on the actions they will do, like changes; we no longer take the initiative to say that this is what we want. I think the community has to buy in to see that this is the need, and we will work with you in this way.” (KII: policy maker 01).

### Selection and development of IV iron implementation strategies

Once the barriers and facilitators to IV iron use to treat anemia in pregnancy were identified in Phase One, Phase Two involved the co-design workshops. The goal of these workshops was to prioritize the identified barriers and facilitators and to develop implementation strategies to address the barriers. The participants developed five strategies, as shown in Table 2. The REVAMP-TT research team discussed and agreed on the most feasible implementation strategies, considering the feasibility and resource capacity of the REVAMP-TT trial. These strategies align with the ERIC domains of developing stakeholder relationships, financial strategy, engaging the community, and training and educating healthcare providers.

1. Develop stakeholder relationships

To address the lack of stakeholder engagement, we presented the study protocol and ongoing progress annually to the Safe Motherhood Technical Working Group and Zomba District Health Management Team for their review, input, and recommendations. Policymakers, government partners, health managers, healthcare providers, institutional maternal health experts, and others who attended the meetings endorsed and appraised the study progress and outcomes.

2. Financial strategies

The intervention costs, including drug procurement, necessary equipment, and additional trial staff recruitment to support the IV iron intervention implementation, were covered in the REVAMP-TT trial by TRUE. This helped to address the barrier to cost for IV iron implementation and resources. 

**Fig. 3:**
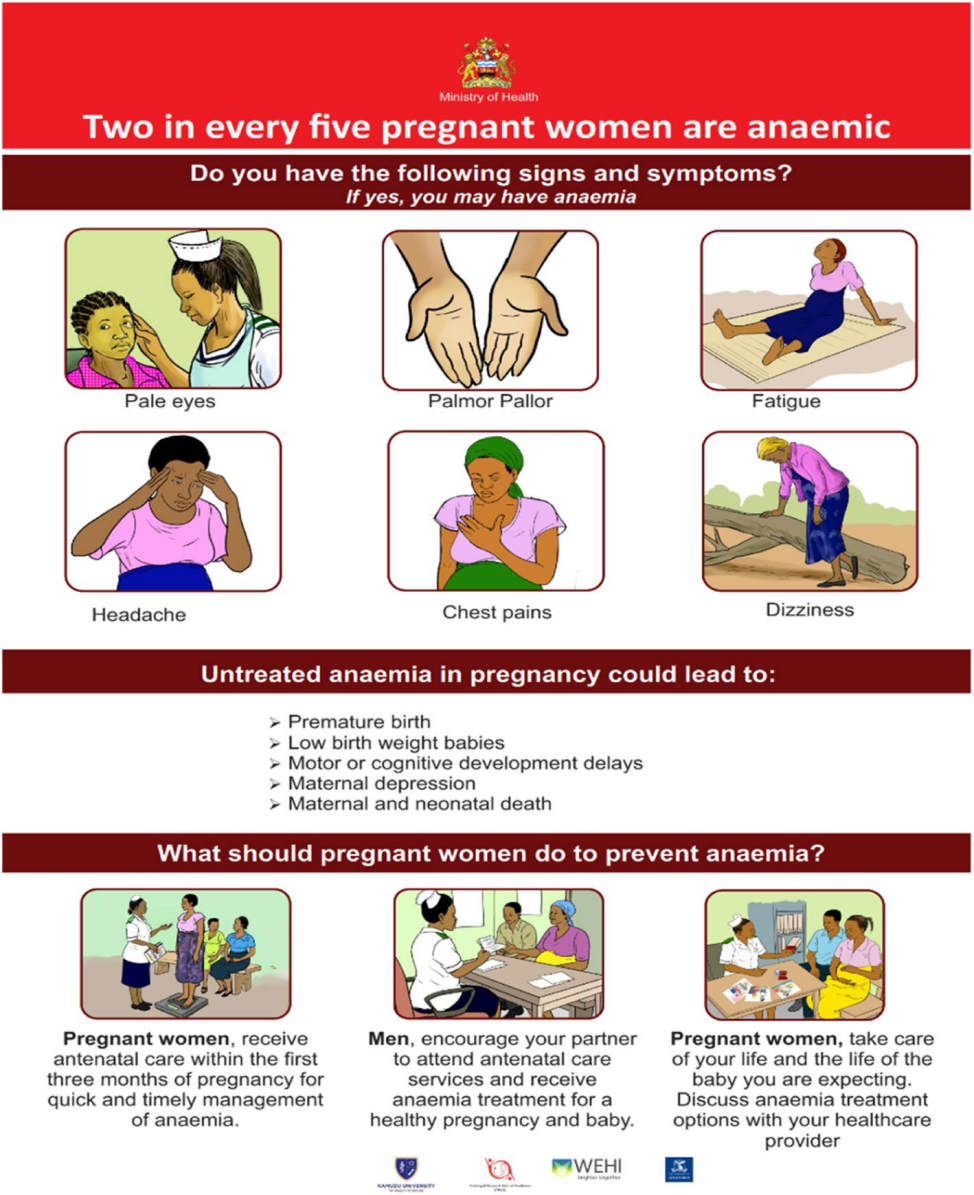
Information, education and communication wallchart for anemia in pregnancy (English Version)

**Fig. 4:**
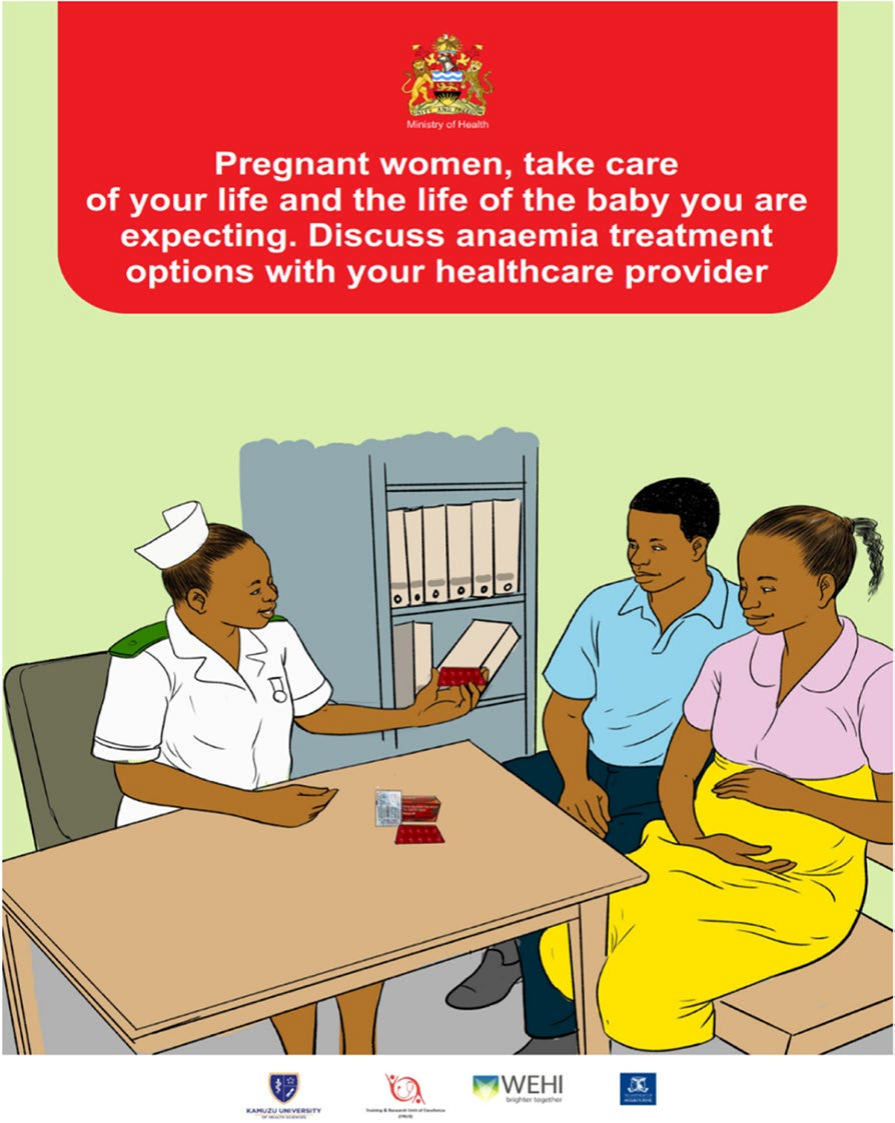
Information, education and communication poster for anemia in pregnancy (English Version)

**Fig. 5:**
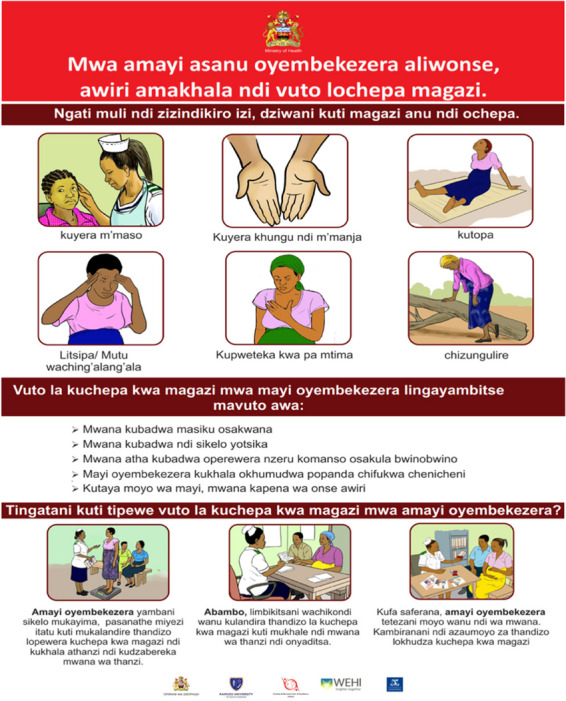
Information, education, and communication wallchart for anemia in pregnancy (Chichewa version)

**Fig. 6:**
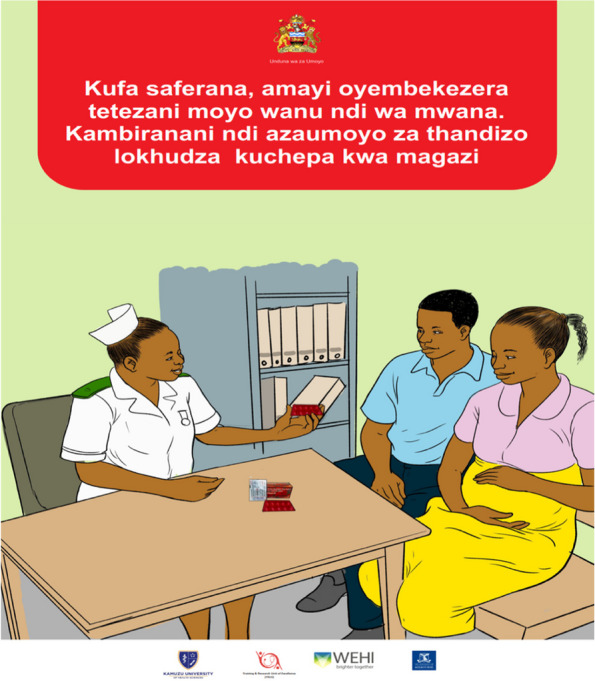
Information, education, and communication poster for anemia in pregnancy (Chichewa version)

3. Engage and educate end-users (pregnant women) and the community
  - Community engagement: To dispel myths and misconceptions, encourage male involvement, and increase knowledge on anemia, we conducted community awareness and sensitization activities highlighting the importance of accessing antenatal care services including anemia screening and treatment in the eight communities surrounding the eight health facilities participating in the REVAMP-TT trial. This involved six meetings at the traditional authority level (e.g. traditional authority leaders, group village heads, village development committees) and one meeting with the Community Health Advisory Group (e.g. local leaders, and community health volunteers). We conducted eight mobile and seven market-day awareness public campaigns in the communities where the eight health facilities participating in the REVAMP-TT trial were located.
  - Development of information, education, and communication (IEC) materials: The co-design workshops revealed a lack of knowledge about anemia among pregnant women, leading to late antenatal care visits, diagnosis, and treatment of anemia. We conducted a national audit that confirmed the absence of anemia in pregnancy IEC materials in Malawi. An international audit of anemia in pregnancy materials informed the creation of IEC material prototypes. In collaboration with the Reproductive Health Department, the Health Education Unit, the Health Promotion Office from the Ministry of Health Malawi, and the graphic designer, we developed an anemia in pregnancy poster and wall chart. Prototypes were pretested with pregnant women and community members (men and women of reproductive age) across the Northern, Central, and Southern regions of Malawi. We conducted twelve focus group discussions, six with pregnant women (10 participants per group) and six with community members (12 participants per group), to assess whether the intended audience understood the key messages and whether the illustrations were appropriate and culturally acceptable. We refined the IEC materials based on feedback from the pregnant women, and community members. We presented the final versions of the poster and wall chart (Figs. 3 and 4 English versions, and Appendix 2, Figs. 5 and 6 for the Chichewa version) to the Zomba District Health Management Team and the Safe Motherhood Technical Working Group. The poster and wall chart materials were endorsed for use at the REVAMP-TT trial sites, aiming to improve pregnant women's knowledge about anemia and encourage anemia screening and timely treatment. Scaling up of the IEC materials nationally is contingent on funding availability in Malawi's health system.

4. Train, educate and support healthcare providers
  a To address the knowledge gap and unfamiliarity with IV iron for healthcare providers, TRUE organized protocol training for the study team on the IV iron profile, preparation, dosage, administration, patient monitoring, managing side effects, and storage of the drug. TRUE also conducted protocol orientation for the healthcare providers at each site involved in the REVAMP-TT trial. Ongoing professional development was ensured throughout the trial implementation period to fulfill the trial-related responsibilities, ensuring high-quality activities and protocol adherence.

## Discussion

Our study identified the barriers and facilitators to IV iron use to treat anemia in pregnancy in the primary healthcare system of Malawi and developed mitigating strategies for the successful implementation of REVAMP-TT. Our key findings highlighted the cost of IV iron, local conditions, local attitude, lack of knowledge, and lack of stakeholder engagement as the barriers to IV iron use. We used data from Phase One (information gathering) to guide the development of strategies in Phase Two (co-design workshops). Specifically, we showed how we mapped the key barriers to the CFIR framework and matched them to the selected strategies in the ERIC compilation. Our results provide insights and suggestions, namely, establishing stakeholder relationships, developing effective community engagement strategies, and supporting healthcare providers to successfully promote the uptake of IV iron successfully implementing the REVAMP-TT trial. We emphasized the need to build strong partnerships with policymakers, government partners, district health managers, healthcare providers, pregnant women, spouses, caregivers, and community leaders to increase uptake and for the successful implementation of REVAMP-TT. More importantly, our methodological approach illustrates how implementation strategies can be developed and provides guidance for researchers seeking to replicate our approach.

### Developing stakeholder relationships: key endeavors for implementation buy-in

Stakeholders are equal members and an integral part of successful health intervention implementation. Deep-rooted relationships with stakeholders can be game changers, fostering collaboration, engagement, and mutual understanding to achieve long-term success [28]. It was evident in our study that the collaborative efforts among various stakeholders allowed the identification of key barriers and facilitators impacting the use of IV iron in the implementation of the REVAMP-TT trial and the development of tailored strategies to create a supportive environment. This ensured the understanding of diverse perspectives to incorporate their input and work together toward a shared common goal in addressing common needs. Through open communication, regular updates, active engagement, and feedback sessions involving policymakers, healthcare providers, and community members, our study gained endorsement, ongoing support, and credibility from stakeholders, enabling a successful environment for implementing the REVAMP-TT trial. For example, developing the IEC materials was successful, due to valuable expertise and insights from stakeholders who were well-versed in the Malawian health system. Likewise, other studies have reported that stakeholder engagement is vital in implementing clinical interventions because it addresses the barriers to implementation, boosts buy-in, and eventually grants acceptability [29–31]. Developing stakeholder relationships fosters collaboration and promotes a sense of ownership, leading to greater commitment and accelerating inclusive decision-making for successful intervention implementation. In implementation science research, establishing collaborative relationships with stakeholders promotes the principles of co-design and co-production and ensures that research findings are easily disseminated and that stakeholders easily support and advocate for the successful adoption of evidence-based practice care [28, 32, 33].

### Developing effective community engagement: strategies to increase intervention adoption

The multifaceted challenges hindering access to antenatal care services, if not resolved, have significant implications for the health of both the mother and the child [34, 35]. For example, we discovered that local attitudes, a lack of male involvement, and a lack of knowledge contribute to pregnant women avoiding accessing antenatal care services, delaying the timely detection and treatment of anemia during pregnancy and consequently accessing IV iron. In Malawian culture, men are household decision-makers, and we found that a lack of male involvement would contribute to decreased demand for IV iron treatment. Involving men in this study ensured that the intervention was respectful, inclusive, and tailored to the community's perceptions. This increased the sense of control and responsibility to embrace the spouse's acceptance of IV iron. Other studies have shown that effective community engagement and male involvement improve health-seeking behaviors and positively impact local attitudes and cultural beliefs [36–43]. It is, therefore, crucial to develop effective community engagement pathways that respect local values and provide open dialogue to enhance knowledge, dispel myths and misconceptions, and overcome resistance.

### Supporting healthcare providers: strategies for implementation success

Supporting healthcare providers is a fundamental strategy for the success of implementing clinical interventions such as IV iron. As highlighted in this study, providing ongoing professional training, sufficient resources, infrastructure, and incentives guarantees the effective delivery of clinical interventions, thereby contributing to improved health system performance. Similarly, investing in enhancing healthcare providers’ capabilities contributes to improved behaviors, attitudes, skills, and knowledge to deliver safe patient-centered care that prioritizes patients’ needs, values, and satisfaction [44, 45]. Other studies have demonstrated that supporting healthcare providers is an effective tool for improving the quality of care and supporting patient health-seeking behaviors. Healthcare providers who are motivated and supported by the system are more committed to their work. Patients trust them and feel motivated and confident to seek medical attention and comply with appointments [46]. While TRUE supported the training and orientation of healthcare providers within the REVAMP-TT trial, it is critical to establish government-funded ongoing training and mentorship programs if the IV iron intervention is to be embedded within routine antenatal care in Malawi. This will ensure that healthcare providers’ skills are up to date and that the delivery of an IV iron intervention remains of high quality.

### Strengths and limitations

This study took a comprehensive approach, integrating perspectives from individual, organizational, and system levels. Using various data collection methods—including interviews, insights from a previous qualitative study, and co-design workshops—we gathered input from policymakers, government partners, health managers, and healthcare providers. We also captured the experiences of pregnant women who had previously received IV iron, along with their spouses, caregivers, community leaders, and members. The iterative process of identifying barriers and discussing facilitators allowed for continuous refinement of implementation strategies, ensuring that diverse perspectives were incorporated. This approach strengthened data triangulation, promoted overall health system improvement, and enhanced the relevance of the study's findings to the local context.

Despite the strengths of the study, there were several limitations. While we have been able to implement the proposed strategies, especially through the development of posters and wallcharts, their effectiveness should be evaluated by observing and interviewing end-users (pregnant women) and healthcare providers to determine their usefulness as educational tools within the antenatal care context. In addition, conducting co-design workshops in one district means that the collection of views represents a small sample. Future studies must consider running co-design workshops in several other districts for further insights. The IV iron intervention was implemented as part of the REVAMP-TT trial using local infrastructure. The intervention costs, including drug procurement, necessary equipment, and additional trial staff recruitment to support the IV iron intervention implementation, were covered by TRUE. This means the costs incurred for procuring medical equipment and supplies, staff training, and supervision are not factored into providing recommendations as part of the evaluation. Future research is required to identify, quantify, and evaluate resources used to deliver an IV iron intervention in routine antenatal care that would help support the Ministry of Health in planning and budgeting decisions as the main program implementers. We acknowledge that providing compensation to participants could raise ethical concerns about undue influence, especially in a population where most individuals do not earn more than $2 per day ​[24]​. Nevertheless, we are obligated to abide by national regulations as described in the consent and ethical clearance section.

## Conclusion

This study demonstrates how an experience-based co-design approach helped to identify key barriers to IV iron use in treating anemia in pregnancy, including cost, resource constraints, knowledge gaps, local attitudes, and lack of stakeholder engagement. It also highlights the process of developing mitigating strategies including financial strategies, strengthening stakeholder relationships, providing training and clinician support, and engaging end-users to ensure the successful implementation of the REVAMP-TT trial. These reflect how key barriers to clinical intervention implementation exist across different levels of the health system and highlight the need to develop tailored multifaceted implementation strategies for effective implementation. The implications of our study are far-reaching as it has demonstrated how the approach enhanced buy-in, ownership and fostered a shared understanding of the intervention goals, and encouraged full participation for the successful implementation of health intervention in real-world settings. Findings from this study enlighten how Malawi and other low-resource settings can make strides in translating research efforts to improve maternal and child health outcomes contributing to. Future studies should assess the effectiveness of the implementation strategies developed in our study to advance knowledge and improve recruitment from both healthcare providers’ and end-users’ perspectives.

## Data Availability

The datasets generated and analyzed during the current study are not publicly available due to participants’ confidentiality.

## Appendix 1

CFIR 2.0 framework domain definition and constructs.

**Table:**

Domain	Definition	Constructs
**Innovation**	The thing being implemented e.g. a new clinical treatment, education program or city service	Intervention source, evidence strength and quality, relative advantage, adaptability, trialability, complexity, design and cost
**Outer setting**	The setting in which the inner setting exists e.g. hospital system, school district or community, that may impact intervention implementation	Critical incident, local attitudes, local conditions, partnership and connections, policies and laws, financing, external pressure.
**Inner setting**	The setting in which the innovation is being implemented that may influence implementation	Structural characteristics, relational connection, communications, culture, tension for change, compatibility, relative priority, incentives system, mission alignment, available resources and access to knowledge and information
**Characteristics of individuals**	The roles and characteristics of individuals involved in implementation that may influence implementation	High level leaders, mid-level leaders, opinion leaders, implementation facilitators, implementation leads, implementation team members, other implementation support, innovation deliverers, innovation recipients, need, capability, opportunity and motivation.
**Implementation process**	The activities and strategies used to implement the innovation that have an influence on the implementation	Teaming, assessing needs, assessing context, planning, tailoring strategies, engaging, doing, reflecting and evaluating

## Appendix 2

Information, education and communication wallchart and poster for anemia in pregnancy (Chichewa version).

## Abbreviations

REVAMP: Randomized controlled trial on the effect of ferric carboxymaltose (FCM) on moderate and severe anemia in pregnant Malawian women in their second trimester
REVAMP-TT: Randomized controlled trial on the effect of intravenous iron on moderate and severe anemia in pregnant women in the third trimester
REVAMP-IS: Randomized controlled trial on the effect of intravenous iron on moderate and severe anemia in pregnant women in the third trimester- Implementation Science
ERIC: Expert Recommendations for Implementing Change
CFIR 2.0: Consolidated Framework for Implementation Research 2.0
IV: IntraVenous
EBCD: Experience-Based Co-Design
SMTWG: Safe Motherhood Technical Working Group
DHMT: District Health Management Team
IEC: Information Education and Communication
